# Molecular and bioinformatics analyses reveal two differentially expressed intracellular GH1 β-glucosidases from the rare alkalophilic fungus *Stachybotrys microspora*

**DOI:** 10.1016/j.gene.2019.04.007

**Published:** 2019-06-30

**Authors:** Salma Abdeljalil, Ines Borgi, Sandra Carvalho, Lamia Jmal-Hammami, Ali Gargouri

**Affiliations:** a Laboratory of Molecular Biotechnology of Eucaryotes, Centre of Biotechnology of Sfax, Road of Sidi Mansour, B.O 1177, 3018, University of Sfax, Tunisia; b Mode of Action Group, Wellcome Centre for Anti-Infectives Research, School of Life Sciences, University of Dundee, Dow Street, Dundee DD1 5EH, Scotland, UK

**Keywords:** GH, glycoside hydrolase, Smbgl1A, *Stachybotrys microspora* β-glucosidase family 1, type A, Smbgl1B, *Stachybotrys microspora* β-glucosidase family 1, type B, EDTA, Ethylenediaminetetraacetic acid, SDS, Sodium Dodecyl Sulfate, *Stachybotrys microspora*, Family 1 β-glucosidases, Differential expression, Secondary structure

## Abstract

The present study reports the isolation and analysis of two novel GH1 β-glucosidases from the alkalophilic fungus *Stachybotrys microspora*, using PCR and Nested-PCR. Three major gene fragments were obtained by PCR: the first two are very similar and constitute a novel gene, which was named *Smbgl1A*, and the third PCR fragment is part of a different gene, named *Smbgl1B*. The truncated gene sequences were completely filled using the recent partial whole genome sequencing data of *S. microspora* (data not yet published).

Moreover, we investigated the relative effects of glucose in comparison to cellulose rather than evaluate their absolute effects.

In fact, RT-PCR analysis showed that while *Smbgl1A* was expressed when the fungus was grown in the presence of cellulose but not when grown with glucose, *Smbgl1B* was equally expressed under both conditions.

The putative catalytic residues and the conserved glycone binding sites were identified. Zymogram analysis showed the intracellular production of β-glucosidases in *S. microspora.* The predicted secondary structure exhibited a classical (β/α)8 barrel fold, showing that both SmBGL1A and SmBGL1B belong to the GH1 family.

Phylogenetic studies showed that SmBGL1A and SmBGL1B belong to the same branch as β-glucosidases from *Stachybotrys chlorohalonata* and *Stachybotrys chartarum*. However, SmBGL1A and SmBGL1B form two distinct clades.

## Highlights

• Isolation of two novel GH 1 β-glucosidases from *Stachybotrys microspora*
• Investigation of the relative effects of glucose in comparison to cellulose
• Zymogram analysis has shown the intracellular production of GH1 β-glucosidases.
• Prediction of the secondary structure with the presence of a classical (β/α) 8 barrel

## 1 Introduction

β-glucosidases are a heterogeneous group of hydrolytic enzymes that are widely distributed in eukaryotic and prokaryotic organisms. These enzymes hydrolyze glycosidic bonds to liberate non-reducing terminal glucosyl residues from glycosides and from short oligosaccharides. β-glucosidases play a variety of pivotal functions in several biological processes, including the degradation of cellulosic biomass and the modification of secondary metabolites. They also play important roles in the regulation of cellulase genes for they represent key enzymes in the synthesis of sophorose, which is an efficient inducer of the cellulolytic system of *Trichoderma reesei* (Singhania et al., 2013).

This group of biologically important enzymes is highly valued in biotechnology as it offers a rich array of enzymes to use in the hydrolysis of β-D-glycoside aroma precursors in juices (Günata et al., 1996), in the debittering of olives through oleuropein hydrolysis (Marsilio et al., 1996), and in the removal of naringin, the glycoside responsible of the bitterness of citrus juice, after its hydrolysis by rhamnosidases (Munish et al., 2011). These enzymes are also valuable for the enhancement of glucose yields from cellulolysis for the subsequent bio-ethanol production process. Last but not least, and thanks to their trans-glucosylation ability, β-glucosidases are useful for the synthesis of several biomolecules with a high added value.

Glycoside hydrolases (GH) have been classified based on their amino acid sequence and structural similarities (Henrissat, 1991). β-glucosidases are the largest group of enzymes in GH families, being present in families GH1, GH3, GH5, GH9, GH30, and GH 116 (Henrissat and Davies, 1997).

GH families exhibiting significant similarities in tertiary structure, catalytic residues, and mechanisms are often grouped into larger structures named “clans”. Families within clans have often been described as having a common evolutionary ancestry (Henrissat and Davies, 1997). Among these clans, GHA has the largest number of families, including the β-glucosidase-containing GH1, GH5, and GH30 families, with GH1 containing the largest number of characterized β-glucosidases (E.C. 3.2.1.21). In addition to having a (β/α)8 barrel structure common to several other hydrolases, glycoside hydrolases of the GHA clan share an active site with two conserved carboxylic acid residues on β-strands 4 and 7 that serve as the general acid (proton donor) and the nucleophile/base, respectively (Henrissat et al., 1995).

Enzymes belonging to the same family also share one of two conserved hydrolysis mechanisms: either the inversion or the retention of the anomeric configuration of the substrate after the cleavage, depending on the spacing between the catalytic residues. It is worth noting that only β-glucosidases retaining the anomeric configuration of the substrate are able to perform trans-glycosylation (Rye and Withers, 2000).

Cellulolytic systems are generally inducible and repressible. The inducers of cellulase production are mainly cellulose (Suto and Tomita, 2001) and its derivatives and other oligosaccharides, disaccharides (sophorose, gentiobiose, laminaribiose, lactose) (Suto and Tomita, 2001; Sehnem et al., 2006), and even monosaccharides like xylose (Suto and Tomita, 2001; Smaali et al., 2004). In contrast, cellulases, as many other hydrolases, are repressed by glucose (Suto and Tomita, 2001) and other simple sugars.

Even though β-glucosidases are considered among cellulases, their inducers are slightly different than those of endoglucanases and cellobiohydrolases. Indeed, it was shown that β-glucosidase activity in *Trichoderma reesei* can be induced by methyl-β-glucoside and less effectively by gentiobiose, while cellobiose did not induce this enzyme (Sternberg and Mandels, 1982). In addition, sophorose (a β-1,2 dimer of glucose) repressed β-glucosidase in this *Trichoderma* QM 6 strain (Sternberg and Mandels, 1982). Another fungus, *Talaromyces amestolkiae*, displayed a different behavior by showing extracellular β-glucosidase activity when grown in various carbon sources of different composition and complexity (1% Avicel Cellulose, glucose, acid slurry from wheat straw, maltose, fructose, glycerol, xylose, cellobiose, or beechwood xylan) (de Eugenio et al., 2017).

The N1 strain is a rare specimen of the *S. microspora* fungus that has the ability to grow on alkaline (pH 9) cellulosic medium and to secrete several β-glucosidases (Amouri and Gargouri, 2006; Saibi et al., 2011; Abdeljalil et al., 2013).

The A19 mutant was isolated after the nitrous acid mutagenesis of this cellulolytic fungal strain and was slightly improved for the production of cellulases (Amouri and Gargouri, 2006).

Considering the promising potential of β-glucosidases from natural origins, the present work reports the isolation of two GH1 β-glucosidases from the rare fungus *S. microspora*, using molecular and bioinformatics studies.

## 2 Materials and methods

### 2.1 Strains, plasmids, and culture conditions

The spores of the mutant strain A19 from *S. microspora* were inoculated into 100 mL of Mandels' liquid medium (Mandels and Weber, 1969) containing 1% of glucose and grown at 30 °C for 5 days, with shaking (150 rpm). The fungal mycelia were harvested by centrifugation.

*Escherichia coli* TOP10F′, *F′ [lacIq Tn10 (Tet*^*R*^*)] mcrA Δ(mrr-hsdRMS-mcrBC) Φ80lacZΔM15 ΔlacX74 recA1 araD139 Δ (ara-leu) 7697 galU galK rpsL (Str*^*R*^*) endA1 nupG* (Invitrogen), was used as a host for plasmid propagation. The pGEM®-T vector (Promega) was used for the cloning of PCR fragments.

### 2.2 DNA isolation

Genomic DNA was isolated from the mycelium of a 5-day-old culture of *S. microspora*, following a modified version of the method previously described by Trigui-Lahiani and Gargouri (2007). Briefly, approximately 1 g of frozen mycelia was grounded to a fine powder with alumina and mixed with 5 mL of extraction buffer [10 mM Tris-HCl (pH 8.0), 50 mM EDTA, and 0.5% sodium dodecyl sulfate (SDS)]. After two extractions with an equal volume of Tris-HCl-phenol, DNA was precipitated overnight with 0.1 volume of 3 M sodium acetate (pH 5.2) and 2.5 volumes of ethanol. The quality and concentration of the DNA were assessed by horizontal gel electrophoresis, on a 0.8% agarose gel and in 50 mM Tris-Acetate EDTA buffer, pH 8. The gel was stained with ethidium bromide and visualized using the UV Trans-illuminator and the Kodak EDAS 290 Electrophoresis Documentation System. Plasmid DNA was isolated from *E. coli* using the alkaline lysis method (Sambrook et al., 1989).

### 2.3 PCR reactions

PCR reactions were performed using 20 pmoles/μL of the designed primers (Table 1), 500 ng of *S. microspora* genomic DNA as template, 0.2 mM dNTP, 2 mM MgCl_2_, 1× Taq buffer, and 0.2 U Taq enzyme. The PCR conditions were as follows: 94 °C for 5 min, 40 cycles of 94 °C for 30 s, 30 s at annealing temperature (50 °C) depending on primer pairs used, 72 °C for 2 min, and a final extension at 72 °C for 7 min. After electrophoresis, PCR products were purified, cloned (named F1–1, F1–2, and F1–3), and sequenced automatically using the BigDye® Terminator v3.1 Cycle Sequencing Kit in the ABI PRISM 3100-Avant Genetic Analyzer, with universal and reverse primers or with internal gene-specific primers.

**Table 1:** Sense (SP) and antisense primers (AP) used for PCR and Nested-PCR.

Primer	Orientation	Nucleotide sequence
P11	SP	5’-TGGGGGTTCGCCACGGCTGCCTAC-3’
P12	SP	5’-AGCATCTGGGACACGTTCTGC-3’
P13	AP	5’-TTCATCACCCTCTTCCACTGG-3’
P14	AP	5’-ATGCGCAAGCAGCTGGGCGAC-3’
P15	SP	5’-TTTTACGGCATGAACCACTAC-3’
P16	AP	5’-TTTTACGGCATGAACCACTAC-3’
P17	AP	5’-TTTGGGGTTACGTATGTGGAT-3’
P18	AP	5’-TTCCCCAAGAAGAGCGCAAAG-3’
A1	SP	5’-CTGGATCACCTTCAACGAGC -3’
A2	SP	5’-TGCAGGGCTACTCGACCGG-3’
A3	SP	5’-GCCCACCTTCACCGAGGAGGAGCG-3’
A4	SP	5’-GAGCGACGCATGCAGTTCCACATT-3’
AS	AP	5′- GTATTTTGAAGATCATCTGG-3’

### 2.4 RNA isolation and expression levels

Total RNA was extracted from *S. microspora* mycelium, grown on glucose- or cellulose-containing culture medium, using TRIzol™ reagent (Invitrogen), according to the manufacturer's instructions. Five micrograms of total RNA were used for reverse transcription using the First Strand cDNA Synthesis Kit (Fermentas), the M-MuLV reverse transcriptase enzyme (Fermentas), and the oligo-dT primer.

The cDNA was submitted to PCR using primers specific to Family 1 of β-glucosidases and β-actin using the following conditions: 94 °C for 5 min, followed by 40 cycles of 94 °C for 30 s, 50 °C for 30 s, and 72 °C for 2 min. An additional extension of 7 min at 72 °C was added to the end of the run. The Family 1 primers used were P12 and P16 for *Smbgl1A* and P12 and P14 for *Smbgl1B*.

The region amplified in the *Smbgl1A* gene using primers P12 and P16 holds an intron; the band obtained had the expected size, confirming that it corresponded to the *Smbgl1A* cDNA. Regarding *Smbgl1B*, since the amplified region had no introns, a control experiment was conducted after DNase addition to ensure that the results of the RT-PCR were not due to the genomic DNA template (data not shown). The β-actin primers used were: 5’-AGCGTGGTATCCTCACGCTC-3′ (sense) and 5’-CTTCATGATGGAGTTGAACG-3′ (antisense). The PCR products were run on a 1% agarose gel and visualized under UV after ethidium bromide staining.

To ensure that the signal intensity was normalized across experiments, agarose gels were incubated with ethidium bromide and photographed using an identical procedure throughout. The intensity of the different bands obtained from agarose gels under glucose or cellulose conditions were measured using GelQuantNET, version 1.8.2, a software used for the quantification of protein, DNA, and RNA in gels (please refer to the website: http://biochemlabsolutions.com/GelQuantNET.html).

### 2.5 Amino acid sequence analysis

The amino acid sequences of SmBGL1A and SmBGL1B were analyzed using InterPro, a program for protein sequence analysis and classification (Hunter et al., 2012).

In fact, InterPro provides functional analysis of proteins by classifying them into families and predicting domains and important sites (http://www.ebi.ac.uk/interpro/).

The catalytic domains of SmBGL1A and SmBGL1B were analyzed to search for Pfam families using the Pfam program, a widely used database of protein families currently containing more than 13,000 manually curated protein families as of release 26.0. (http://pfam.sanger.ac.uk/) (Finn et al., 2014).

### 2.6 Multiple alignment of amino acids sequences and domain modeling

Multiple alignment of SmBGL1A and SmBGL1B with known fungal β-glucosidase amino acid sequences was performed using ClustalW of the BioEdit Package (v.7.0.5). The secondary structures of the deduced amino acid sequences were predicted by homology modeling using the Swiss-Model Server (Arnold et al., 2006), using the crystal structure of ThBgl2 from *Trichoderma harzianum* (PDB: 5jbo.1A) (Florindo et al., 2018) as a template for SmBGL1A and the crystal structure of GH1 β-glucosidase from *Humicola insolens* (PDB: 4mdo.1A) for SmBGL1B. The constructed models were visualized with the Swiss-Pdb Viewer software v. 4.01 (Guex and Peitsch, 1997). The parameters and prediction quality of the modeled structures were evaluated using the ProSA server (Wiederstein and Sippl, 2007).

### 2.7 Extraction of intracellular proteins and zymogram analysis

*S. microspora* was cultivated under glucose or cellulose conditions in 500-mL Erlenmeyer flasks containing 100 mL of Mandels' medium supplemented with 100 μg/mL ampicillin to avoid bacterial contamination, for 3 days at 30 °C at 150 rpm rotary agitation. One gram of wet cells was re-suspended in 10 mL of citrate buffer (50 mM pH 5.5, 1 mM PMSF, and 1 mM EDTA) and sonicated on ice at 40% amplitude for 20 min (10 cycles of 10 s, followed by 10 s off intervals). The lysate was centrifuged at 13,000 rpm for 30 min, and the supernatant was recovered and processed for zymogram analysis.

Zymogram analysis of β-glucosidase activity was performed by adding loading buffer to the supernatant samples and subjecting them to sodium dodecyl sulfate-polyacrylamide gel electrophoresis (SDS-PAGE; carried out using a 5% stacking gel and a 10% separating gel) without boiling prior to loading (Laemmli and Favre, 1973). After electrophoresis, the gel was incubated for 2 h in Tris-HCl 20 mM, pH 8.0, to remove SDS, allowing the renaturation of proteins. After 15 min of equilibration in 50 mM sodium acetate buffer, pH 5.0, a diluted solution of 25 μg/mL MUG (4-Methylumbelliferyl β-D-glucopyranoside) was added to gel, and this was visualized under UV light, with excitation at 366 nm and emission at 445 nm. The location of the activity is indicated by the fluorescence emitted by the 4-Methylumbelliferone released *via* enzyme action.

### 2.8 Phylogenetic analyses

The phylogenetic tree of fungal β-glucosidases was constructed using the software PhyML, estimating the maximum likelihood (ML) phylogenies from alignments of nucleotide or amino acid sequences (Guindon and Gascuel, 2003).

Phylogenetic analyses were performed using the phylogeny.fr platform (http://www.phylogeny.fr/) with the “A la Carte” mode: Muscle for multiple alignment, PhyML for tree building, and TreeDyn for tree rendering.

## 3 Results and discussion

### 3.1 Primer design and isolation of family 1 β-glucosidase gene fragments

The amino acid sequences of several Family 1 β-glucosidases were initially aligned to reveal consensus regions so that the amplification of Family 1 β-glucosidase DNA fragments in *S. microspora* could be directed to the corresponding genomic sequences.

As the 18 S rDNA sequence of *S. microspora* showed significant homology with that of *T. reesei*, the primers were, therefore, designed according to the nucleotide sequence of the *bgl2* gene that encodes a Family 1 β-glucosidase of *T. reesei* (Table 1).

In fact, the two sequences share about 96% of identity, and the phylogenetic studies of 18S rRNA gene variation among common airborne fungi have shown that *T. reesei* and *S. microspora* are closely related, belonging to the same clade (Wu et al., 2003). These primers were used to perform PCR and Nested-PCR of the genomic DNA of *S. microspora*.

Since PCR reactions did not yield any amplification, Nested-PCR was performed using all consensus primer combinations. The resulting amplicons had the expected sizes, according to the *T. reesei bgl2* gene, and were purified from the agarose gel (Table 2). Three fragments were directly sequenced and analyzed using the Basic Local Alignment Search Tool (BLAST; last version released: BLAST+ 2.8.1; www.ncbi.nlm.nih.gov/). The analysis revealed a significant degree of similarity with Family 1 of β-glucosidases.

**Table 2:** Analysis of fragments amplified by PCR and Nested-PCR. The observed size of fragments were estimated according to a molecular-weight size marker.

Primers (PCR)	Primers (NESTED-PCR)	Amplified	Expected size of fragment(bp)	Observed size of fragment(bp)
(P11,P16)	(P12,P14)	F 1–1	734	800
(P11,P17)	(P12,P16)	F 1–2	809	850
(P11,P17)	(P15,P17)	F 1–3	423	500

### 3.2 Sequence analysis

**Fig. 1:**
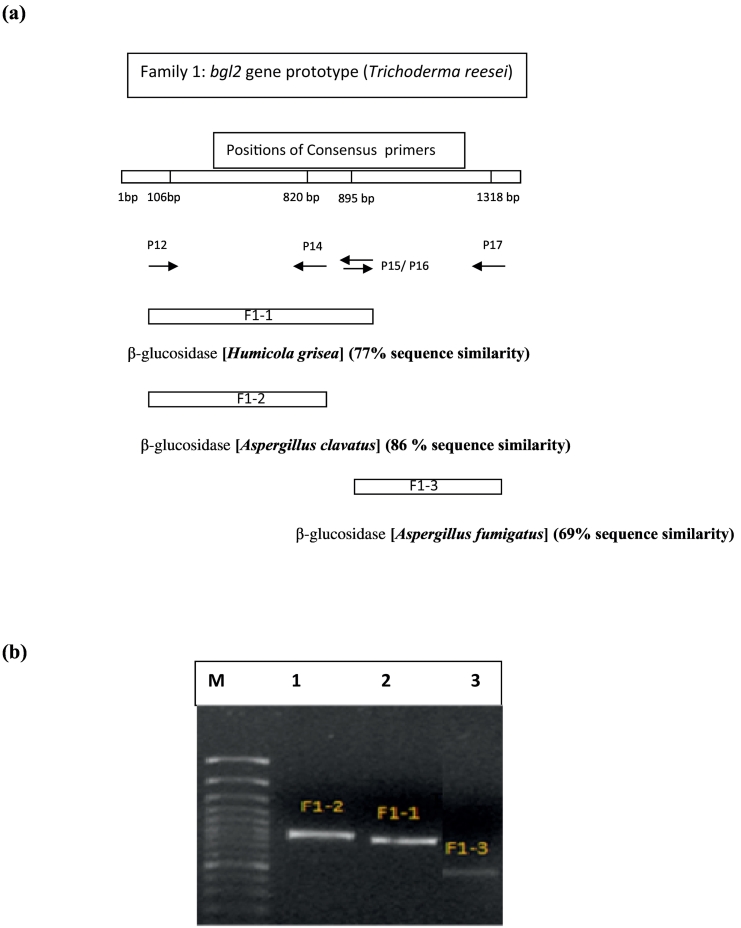
(a) Position of *Smbgl1A* and *Smbgl1B* genes with consensus primers according to the *bgl2* gene from *T. reesei*. (b) Nested-PCR analysis of Family 1 β-glucosidase genes. Lane M: 1 kb DNA Ladder; Lane 1: fragment F1–1 amplified with primers P12 and P14; Lane 2: fragment F1–2 amplified with primers P12 and P16; Lane 3: fragment F1–3 amplified with primers P15 and P17.

The F1–1 sequence showed high homology with the gene coding for the thermostable β-glucosidase of *Humicola grisea* (*bgl4*) (Fig. 1). The enzyme encoded in this gene was previously purified and reported to exhibit two other activities: β-galactosidase and β-fucosidase (Peralta et al., 1990).

F1–2 and F1–3 fragments displayed a high degree of homology with β-glucosidases from *Aspergillus* species (Fig. 1), which are known for their ability to degrade plant polysaccharides (De Vries, 2003; De Vries et al., 2000).

According to known fungal genes, F1–1 and F1–2 fragments correspond to the N-terminal domain of β-glucosidases. However, their alignment did not show a high degree of similarity (52% of homology; Bioedit software), suggesting that they belong to two different genes. Moreover, findings from the NetAspGene 1.0 Server, a splicing site prediction program for the genus *Aspergillus* (Wang et al., 2009), revealed that, while F1–1 did not have introns, F1–2 contained one intron. The amino acid sequence encoded by the F1–3 fragment corresponded the C-terminal domain and displayed strong homology with the β-glucosidase from *Aspergillus fumigatus* (Fig. 1). Analysis of its nucleotide sequence by homology with other Family 1 β-glucosidase genes showed an intron and two splicing sites, which were also confirmed by NetaspGene and FSPLICE 0.1 (data not shown).

### 3.3 Determination of the number of isolated β-glucosidase genes

The results of bioinformatics analyses revealed that while F1–2 and F1–3 fragments had high homologies with the β-glucosidase from *Aspergillus*, approximately 86% and 69% respectively, the F1–1 fragment showed significant similarity with the β-glucosidase from the thermophilic fungus *H. grisea*, approximately 77%. These results suggested that while F1–2 and F1–3 could belong to the same gene, F1–1 derives from a distinct gene.

PCRs using specific primers located within the various isolated fragments were, therefore, performed to verify the validity of this hypothesis.

Amplification of a fragment was observed when a sense primer annealing in F1–2 and an antisense primer annealing in F1–3 were used (data not shown), confirming the hypothesis that F1–2 and F1–3 are fragments of the same gene. This gene was designated *Smbgl1A*. No amplification was observed when using a primer specific for F1–1 and a primer specific for F1–2 or F1–3, showing that F1–1 is a fragment of a different gene, which was designated *Smbgl1B*. The genome of *S. microspora* is under sequencing (*i.e.* not yet finished), which contributed to the truncated gene sequences at 5′ and 3′ ends.

All the fragments obtained by PCR (F1–1, F1–2, and F1–3) were aligned to contigs from the partial genome sequence and identified as *Smbgl1A* (containing F1–2 and F1–3) and *Smbgl1B* (containing F1–1). A manuscript reporting the finalized results of this work is in preparation.

### 3.4 Codon usage analysis

An analysis from a genomic perspective revealed an average GC content of 57% in nuclear genes of *Neurospora crassa* and confirmed prior findings that suggested a bias of a cytosine base at the third position in *N. crassa* codons (Radford and Parish, 1997); the same result was found in the codons of *Smbgl1B* of *S. microspora*, while guanine was the preferred base in the codons of *Smbgl1A* (Table 3).

**Table 3:** Codon usage in *S. microspora* Smbgl1A (MH036528), Smbgl1B (MH036529) and Smbgl3 (JQ417861) genes assessed by using the online Codon Usage Calculator (www.biologicscorp.com/tools/CodonUsageCalculator/#.W2Nl99JKjIU).

Smbgl1A	Smbgl1B	Smbgl3	Smbgl1A		Smbgl1B	Smbgl3	Smbgl1A		Smbgl1B	Smbgl3	Smbgl1A		Smbgl1B	Smbgl3
UUU F 13	4	13	UCU S	2	4	9	UAU Y	9	4	13	UGU C	1	2	3
UUC F 10	21	24	UCC S	6	10	12	UAC Y	16	23	21	UGC C	7	4	4
UUA L 3	0	1	UCA S	4	0	7	UAA *	0	0	0	UGA *	0	0	0
UUG L 9	7	9	UCG S	6	1	13	UAG *	1	1	1	UGG W	14	15	22
CUU L 5	9	15	CCU P	4	9	16	CAU H	5	3	6	CGU R	4	9	12
CUC L 5	10	20	CCC P	10	17	23	CAC H	6	7	9	CGC R	4	12	12
CUA L 5	0	6	CCA P	4	0	11	CAA Q	1	1	13	CGA R	10	2	11
CUG L 11	11	15	CCG P	9	2	7	CAG Q	14	11	27	CGG R	9	0	3
AUU I 11	6	15	ACU T	0	6	16	AAU N	7	2	17	AGU S	4	2	3
AUC I 10	18	21	ACC T	5	19	14	AAC N	8	21	33	AGC S	4	8	16
AUA I 4	0	3	ACA T	8	0	15	AAA K	3	5	5	AGA R	5	0	6
AUG M 6	5	15	ACG T	7	1	8	AAG K	12	22	9	AGG R	5	2	5
GUU V 4	2	17	GCU A	12	11	23	GAU D	14	12	28	GGU G	2	15	32
GUC V 6	19	26	GCC A	8	17	24	GAC D	21	24	30	GGC G	19	16	43
GUA V 4	0	5	GCA A	7	1	8	GAA E	19	2	13	GGA G	10	4	15
GUG V 5	3	10	GCG A	12	2	16	GAG E	21	29	28	GGG G	11	5	6

Moreover, the analysis revealed an average GC content of 54% and 57% GC for *Smbgl1A* and *Smbgl1B*, respectively.

The study of 45 highly or poorly expressed genes in *Aspergillus nidulans* indicated that, although the GC content of the genome is close to 50%, the codon usage is highly biased to approximately 20 “optimal codons” that are characterized by ending in C or G (Lloyd and Sharp, 1992). The analysis of *Smbgl3*, a Family 3 β-glucosidase gene that we have previously isolated (Abdeljalil et al., 2013), revealed a bias toward C and G bases at the third position. The same result was found for *Smbgl1B*; regarding the *Smbgl1A* gene, there was a preference for A and T (Table 3).

The effects of codon usage bias on gene expression were previously thought to be due to the impacts on translation as there is a correlation between codon frequency and tRNA concentration, a kind of signature in each species (Kimura, 1968, Kimura, 1984). In fact, codon usage bias strongly correlates with protein and mRNA levels genome-wide in the filamentous fungus *Neurospora*.

Zhou et al. (2016) found that the impacts of codon usage on gene expression are mainly due to effects on transcription and not only on translation.

Together, these results have uncovered an unexpected role for codon bias in determining transcription levels by affecting chromatin structures and suggest that codon bias is the result of genome adaptation to both transcription and translation machineries (Zhou et al., 2016).

Concerning *S. microspora*, differences in codon usage between *Smbgl1A* and the other β-glucosidase genes, *Smbgl3* and *Smbgl1B*, led us to hypothesize that it was expressed differentially in relation to the other two genes.

### 3.5 RNA isolation and expression levels

The differential expression of β-glucosidases in *S. microspora* was investigated in previous biochemical studies (Saibi et al., 2011).

Many carbon sources were tested, namely glucose, xylose, galactose, cellobiose, lactose, sucrose, glycerol, sorbitol, CMC, Avicel cellulose, wheat bran, gruel, and sugarcane bagasse. Mandels' medium without any carbon source was used as control. All substrates induced β-glucosidase production except glycerol, which could be, therefore, considered a non-inducing carbon source. Glycerol gave the same result as the control medium without any carbon source. However, the highest activity was curiously obtained using glucose, as it is generally known as a universal repressor of the majority of hydrolases (Saibi et al., 2011).

According to these previous biochemical studies, we limited the current study to glucose and cellulose.

**Fig. 2:**
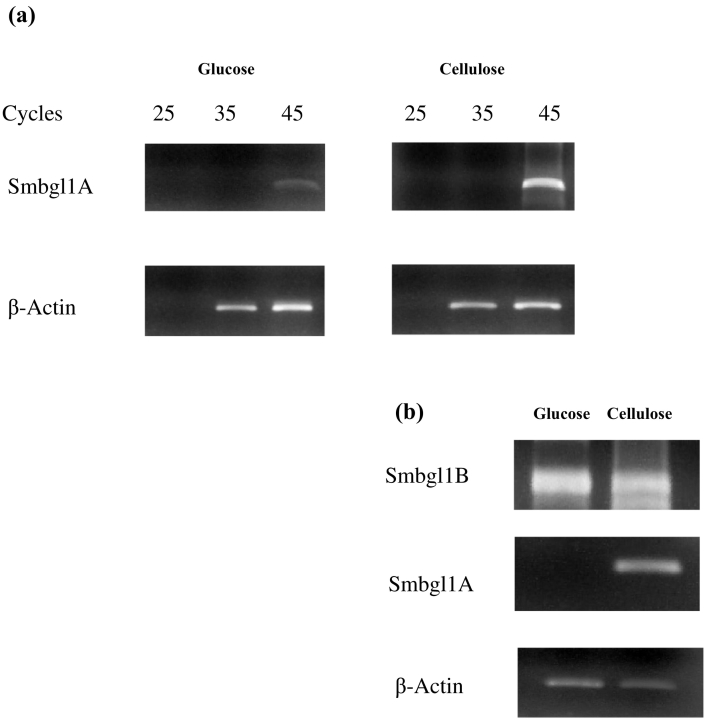
(a) RT-PCR analysis of Family 1 β-glucosidase mRNAs using 25, 35, and 45 cycles, with primers P12 and P16. (b) RT-PCR analysis of Family 1 β-glucosidase mRNAs using 40 cycles and primers P12 and P16 for *Smbgl1A* and P12 and P14 for *Smbgl1B*. mRNAs were extracted from an A19 culture grown in the presence of glucose or cellulose. The actin transcript was used as an internal control. RT-PCR products were analyzed by electrophoresis on 1% agarose gel.

RT-PCR experiments were performed on RNA extracted from *S. microspora* grown in 1% glucose or 1% cellulose. A semi-quantitative analysis was performed by assessing the RT-PCR results obtained at 25, 35, and 45 cycles. The amplified *Smbgl1A* fragment was observed only after 45 cycles and was noted to be better expressed on cellulose- than on glucose-based media (Fig. 2a). The β-actin transcript, used as an internal control, was more abundant, since it was detected after 35 cycles of PCR (Fig. 2a). Fig. 2b shows a clear difference between the two conditions at cycle 40: the absence of the amplified fragment on glucose-based culture *versus* its presence on the cellulose-based one. *Smbgl1A* gene expression was similar to that of the cellobiose-hydrolyzing *T. reesei bgl2* (Saloheimo et al., 2002) and *Phanerochaete chrysosporium bgl1B* (Tsukada et al., 2006).

Interestingly, and contrary to the cellulose-induced expression of *Smbgl1A*, *Smbgl1B* was expressed in the presence of glucose, showing a slightly stronger response than in the presence of cellulose (Fig. 2b), a behavior that is not commonly reported for cellulolytic genes. SmBGL1B shows a high degree of sequence identity with a glucose-tolerant GH1 β-glucosidase from *Humicola insolens* (De Giuseppe et al., 2014).

The program GelQuantNET was used to measure the intensity of the different bands obtained from agarose gels. Regarding *Smbgl1B*, the bands had as intensity values 88,120,989.9 for the presence of glucose and 68,186,131.3 for cellulose.

However, for *Smbgl1A*, which was expressed only in the presence of cellulose, the corresponding band intensity was approximately 1,696,266.0. The intensities of the actin bands were 2,070,480.0 and 1,654,998.0 under glucose and cellulose conditions, respectively.

### 3.6 Study of the amino acid sequences of SmBGL1A and SmBGL1B

The results obtained with the Bioedit program revealed that the amino acid sequences of SmBGL1A and SmBGL1B are 50% identical. The residues involved in catalysis and in glycone binding are conserved in both sequences.

It is worth noting in this context that crystal structures of several GH1 enzymes were previously determined and substantial data were reported on GH1 reaction mechanism (retaining), glycone binding site (subsite −1), and aglycone binding site (subsite +1) (Nijikkena et al., 2007).

The retaining mechanism of GH1 enzymes was previously reported to involve two conserved glutamate residues: a nucleophile and a proton donor that are 5 Å distant from each other (Isorna et al., 2007).

**Fig. 3:**
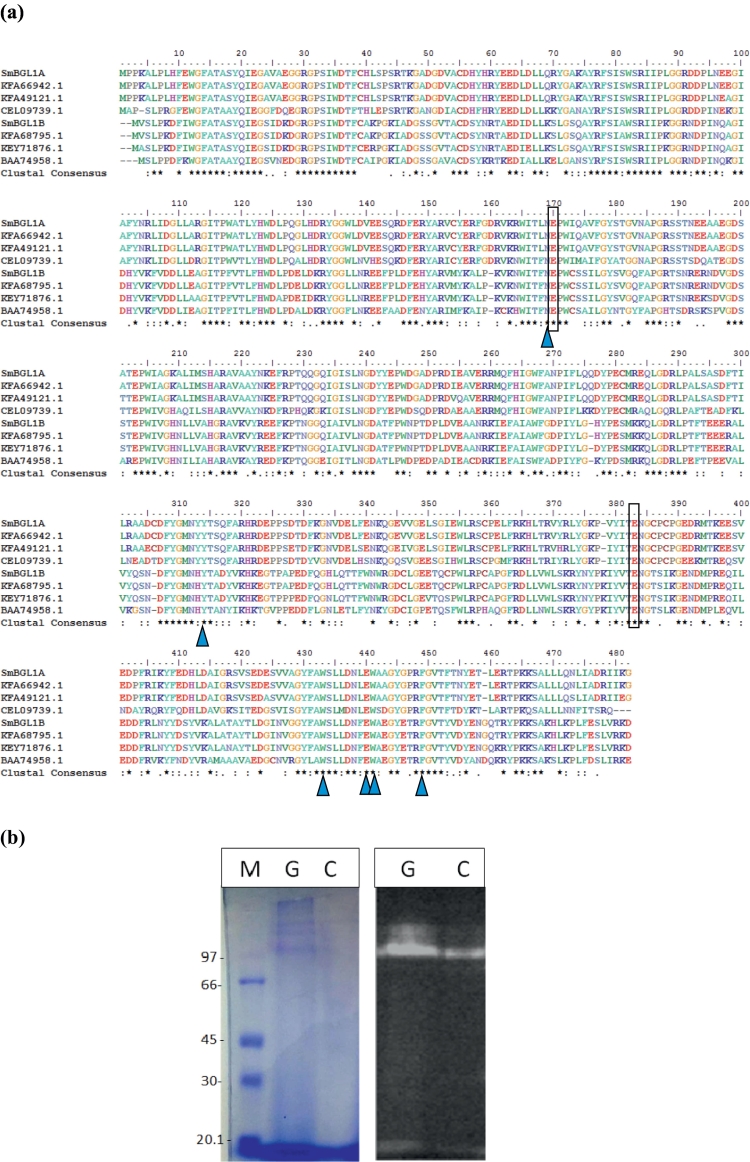
(a) ClustalW alignment of SmBGL1A (GenBank acc. no.: MH036528) and SmBGL1B (MH036529) from *Stachybotrys microspora* with known fungal β-glucosidase amino acid sequences: *Stachybotrys chlorohalonata* (KFA66942.1 and KFA68795.1), *S. chartarum* (KFA49121.1 and KEY71876.1), *Aspergillus calidoustu*s (CEL09739.1), and *Humicola grisea* (BAA74958.1). The highly conserved catalytic residues are boxed. The residues involved in glycone binding sites are indicated by blue triangles. (b) Zymogram analysis of intracellular β-glucosidases under non-denaturing conditions. G: glucose-containing media; C: cellulose-containing media. The image on the left is a blue Coomassie-stained gel ran under the same conditions. M: Protein Molecular Weight Marker. (For interpretation of the references to colour in this figure legend, the reader is referred to the web version of this article.)

In the present work, several known structures of β-glucosidases and those presenting significant homologies with SmBGL1A and SmBGL1B were submitted to a multiple alignment (Fig. 3a), and the findings revealed that the acid/base and catalytic nucleophile were conserved in all GH1 enzymes.

Structural data and the analysis of the enzyme-ligand hydrogen bonding interactions have allowed the identification of the catalytic residues for Family 1 β-glucosidases (Marana, 2006).

Interestingly, the TXNEP motif was identified as consisting of highly conserved residues surrounding the acid/base residue, with X referring to a hydrophobic amino acid residue (Marana, 2006).

Some residues were shown to be important for and conserved in glycone binding in the GH1 β-glucosidases. The extensive interactions at the glycone binding site (subsite −1) are responsible for the strict substrate specificity of BGL1A from *P. chrysosporium* (De Vries, 2003).

Almost all these residues were conserved among all GH1 β-glucosidases (Fig. 3a). However, the degree of conservation in the aglycone binding site (subsite +1) was lower than that in the glycone binding site, which explains the differences recorded in terms of substrate specificity of the enzymes (Marana, 2006). Furthermore, InterPro analysis of SmBGL1A and SmBGL1B revealed that they contain the domain IPR013781 that represents the catalytic TIM beta/alpha barrel common to many different families of glucosyl hydrolases.

The catalytic domains of SmBGL1A and SmBGL1B were used in a Pfam search that resulted in a significant Pfam-A match with clan CL0058. This large superfamily contains a range of glucosyl hydrolase enzymes that possess a TIM barrel fold; among them is the Family Glyco_hydro_1, PF00232. Accordingly, the catalytic domain of SmBGL1A and SmBGL1B belongs to the family PF00232.

The theoretical pI and Mw for SmBGL1A and SmBGL1B were calculated as 4.97 and 55 kDa and 5.24 and 54 kDa, respectively (https://web.expasy.org/).

The analysis of the amino acid composition for SmBGL1A revealed a total of 75 negatively charged residues (Asp + Glu) and 52 positively charged residues (Arg + Lys). The instability index (II) was computed to be 42.70, thus classifying the protein as unstable (https://web.expasy.org/).

Regarding SmBGL1B, it has 67 negatively charged residues (Asp + Glu) and 52 positively charged (Arg + Lys). The instability index (II) was computed to be 31.07, classifying the protein as stable.

More importantly, SmBGL1A and SmBGL1B were predicted to have an intracellular location in their soluble form (DeepLoc-1.0 server), due to the absence of signal peptide (SignalP 4.0 Server).

Previous biochemical studies in the same strain have shown a particularly secreted enzymatic activity of β-glucosidase when grown on glucose as the sole carbon source (Saibi et al., 2011) and a constitutive extracellular β-glucosidase produced in glucose and cellulose conditions (Abdeljalil et al., 2013).

To study the predicted intracellular location of SmBGL1A and SmBGL1B, we performed a zymogram analysis of intracellular lysates from fungal mycelia (Fig. 3b). The results indicate the presence of β-glucosidase activities under glucose and cellulose conditions, confirming the intracellular location of the studied genes.

Due to the similar predicted molecular weights of SmBGL1A and SmBGL1B (55 kDa and 54 kDa, respectively), only a single band could be observed in glucose-grown cultures. However, on zymography analysis (Fig. 3b), both β-glucosidases seemed to have molecular weights higher than the predicted ones.

Since, for zymography, lysates were not heated prior to gel electrophoresis, the enzymes were not completely denaturated, which could explain the difference between predicted and observed molecular weights.

### 3.7 Prediction of the secondary structure of SmBGL1A and SmBGL1B

SmBGL1A showed 73.94% sequence identity with ThBgl2 from *Trichoderna harzianum*, whose crystallization and 3D structure determination were recently performed (Florindo et al., 2018). This 3D structure (PDB: 5jbo.1A) was, therefore, used as a template to build a model for SmBGL1A.

**Fig. 4:**
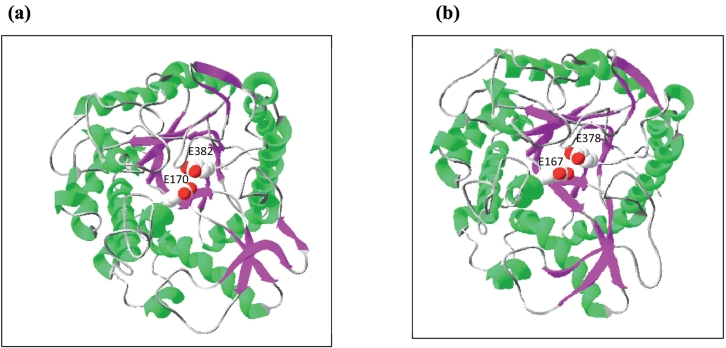
Structural modeling of SmBGL1A (a) and SmBGL1B (b) using the templates 5jbo.1A and 4mdo.1A, respectively, generated by the SwissModel automated mode and 3D-visualization with Swiss-Pdb Viewer 4.0. The two catalytic glutamate residues are indicated in the two constructed models.

SmBGL1B showed 75.21% sequence identity with a GH1 β-glucosidase of *H. insolens*, whose 3D structure (PDB: 4mdo.1) was determined by De Giuseppe et al. (2014). The two models (Fig. 4) presented a monomeric form with a single (β/α)8 barrel domain, a structural feature conserved in all GH1 enzymes (www.cazy.org). The acid/base and catalytic nucleophile glutamate residues were conserved in their positions at the end of the β4 strand and at the end of the β-strand 7, respectively (Fig. 4).

The parameters and prediction quality of the modeled structures were evaluated using ProSA server (Wiederstein and Sippl, 2007). The overall model quality gave a Z score of −9, 5 for SmBGL1A and − 10.62 for SmBGL1B. The determined scores are within the range of native proteins of similar size (data not shown).

### 3.8 Phylogenetic analyses

Standard Blastp (protein-protein BLAST; www.ncbi.nlm.nih.gov) was used for retrieving GH1 amino acid sequences. The algorithm parameters were modified for a value of 250 for “Max target sequences” for both SmBGL1A and SmBGL1B.

Phylogenetic analysis was performed on the phylogeny.fr platform. Sequences were aligned with MUSCLE (v3.8.31). After alignment, ambiguous regions containing gaps or poorly aligned were removed using Gblocks (v 0.91b). The phylogenetic tree was constructed using the maximum likelihood method implemented in the PhyML program (v3.1/3.0 alRT). The Jones-Taylor-Thornton matrix (protein) was selected with the gamma shape parameter estimated directly from the data (gamma = 0.872). The reliability for internal branch was assessed using the bootstrapping method (500 bootstrap replicates).

**Fig. 5:**
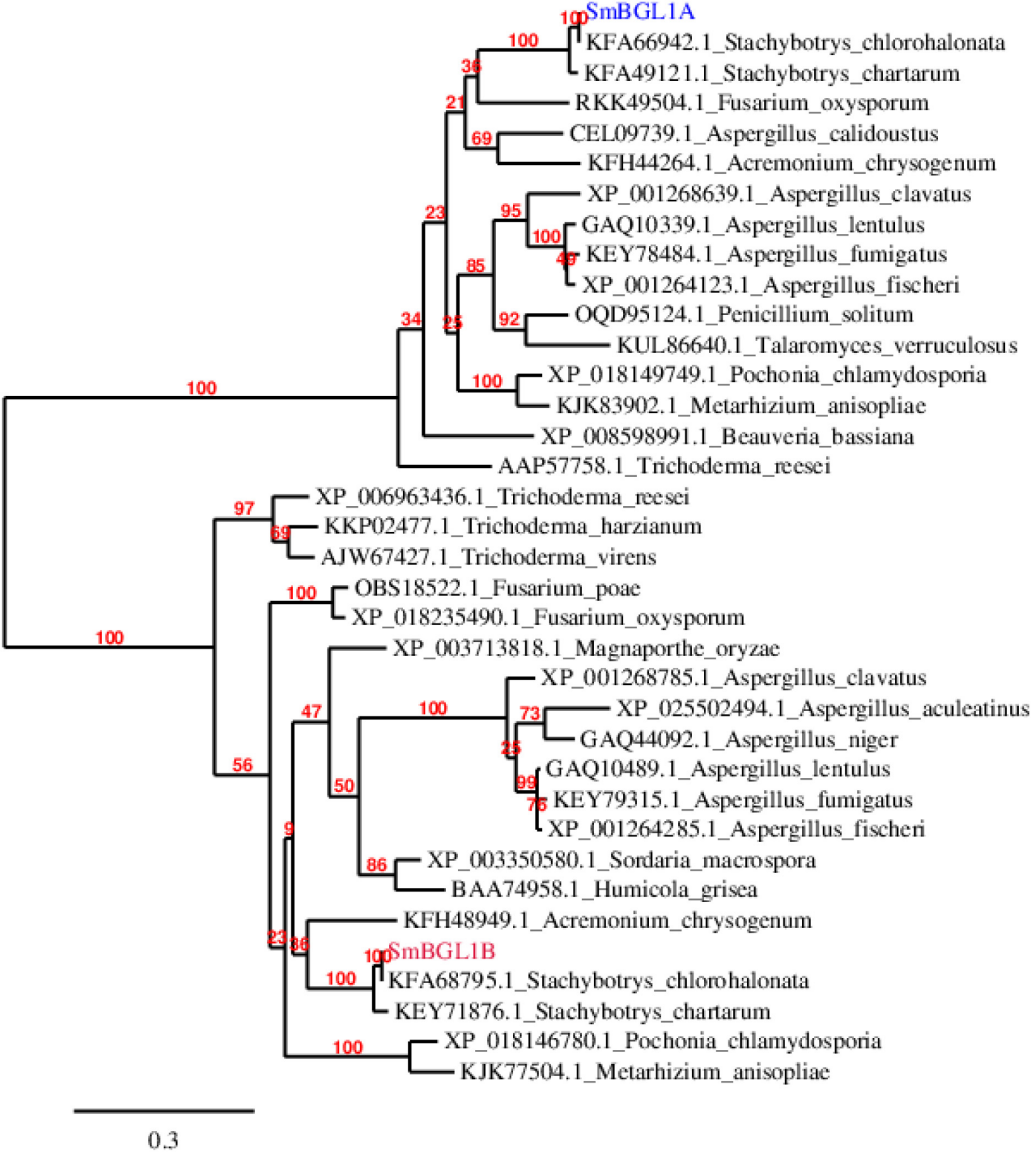
Phylogenetic tree constructed with the phylogeny.fr platform, using PhyML for tree building based on the amino acid sequences of fungal β-glucosidases. Branch-support values calculated by bootstrapping procedure are indicated in red. (For interpretation of the references to colour in this figure legend, the reader is referred to the web version of this article.)

Each of SmBGL1A and SmBGL1B formed a single clade with β-glucosidases from the two ascomycete fungi *Stachybotrys chlorohalonata* and *Stachybotrys chartarum*, which was distinct from *Trichoderma* and *Aspergillus* species (Fig. 5).

Analysis of the phylogenetic tree showed that SmBGL1A sequence is more closely related to *Aspergillus clavatus* than *Metharhizium anisopliae* and *Beauveria bassiana*. However, SmBGLB is closely related to the two fungi *Metarhizium anisopliae* and *H. grisea*.

Regarding intracellular β-glucosidases from *T. reesei*, Xu et al. (2014) studied CEL1a and CEL1b, which are essential for cellulase induction in the presence of lactose. The authors mention that the mechanism underlying the induction is, however, not fully understood. They investigated the cellular functions of CEL1a and CEL1b in the induction of cellulase genes by lactose in *T. reesei* and showed that CEL1a and CEL1b are functionally equivalent in mediating the induction but the simultaneous absence of these intracellular β-glucosidases abolishes *cbh1* (cellobiohydrolase) gene expression under lactose conditions(Xu et al., 2014).

According to the phylogenetic analysis, SmBGL1A and SmBGL1B and β-glucosidases from *Trichoderma* species belong to different clusters (Fig. 5), and we should recall here that SmBGL1B showed significant homology with β-galactosidases (or lactases).

Moreover, SmBGL1B showed high homology with the thermostable β-glucosidase of *H. grisea* BGL4, which has two other activities: β-galactosidase and β-fucosidase (Takashima et al., 1999).

Saloheimo et al. (2002) characterized an intracellular β-glucosidase enzyme, BGLII (CEL1a), and its gene (*bgl2*) from the cellulolytic fungus *T. reesei* (*Hypocrea jecorina*). The expression pattern of *bgl2* is similar to that of other cellulase genes known from this fungus, and the gene would appear to be under the control of carbon catabolite repression mediated by the *cre1* gene.

According to our results, SmBGL1A, which clusters distantly with *T. reesei* β-glucosidases (Fig. 5), showed a similar expression profile to BGLII, being induced in cellulose-containing media and repressed in the presence of glucose. Therefore, there is no evident relation between the transcriptional profile and protein function regarding how (and why) SmBGL1A and SmBGL1B belong to a group rather than the other. Compared to BGL4 from *H. grisea*, recombinant BGLII showed a weak β-galactosidase activity.

However, we have shown that SmBGL1A and SmBGL1 B belong to two distinct clades.

## 4 Conclusion

We isolated two novel Family 1 β-glucosidases from the alkalophilic fungus *Stachybotrys microspore*, using PCR and Nested-PCR techniques. The truncated gene sequences from *Smbgl1A* and *Smbgl1B* were filled using the whole genome sequencing data of *S. microspora*. Genomic sequence analysis shows a codon bias toward G/C at the third position, and primary and secondary structure analyses suggest that SmBGL1A and SmBGL1B are intracellular enzymes and have the classical (β/α) 8 barrel of the GH1 family.

Moreover, the relative effects of glucose and cellulose on the transcript levels of *Smbgl1A* and *Smbgl1B* were addressed, and RT-PCR analysis showed that *Smbgl1A* is expressed in cellulose-containing medium and not under glucose conditions, while *Smbgl1B* is expressed under both conditions.

Zymogram analysis confirmed the intracellular production of SmBGL1A and SmBGL1B β-glucosidases.

Phylogenetic analyses show that SmBGL1A and SmBGL1B belong to two distinct clades, with each β-glucosidase being close, in each clade, to those of the two ascomycete fungi *S. chlorohalonata* and *S. chartarum*.

## Declaration of interests

The authors declare that they have no known competing financial interests or personal relationships that could have appeared to influence the work reported in this paper.

The authors declare the following financial interests/personal relationships which may be considered as potential competing interests.
